# Predictive model of chemotherapy-related toxicity in elderly Chinese cancer patients

**DOI:** 10.3389/fphar.2023.1158421

**Published:** 2023-04-26

**Authors:** Yuwei Hua, Yuling Zou, Mei Guan, Hsiang-Yu Yuan, Yanping Zhou, Fengshuo Liu

**Affiliations:** ^1^ Department of Oncology, Peking Union Medical College Hospital, Chinese Academy of Medical Sciences and Peking Union Medical College, Beijing, China; ^2^ Department of Biomedical Sciences, City University of Hong Kong, Hong Kong SAR, China

**Keywords:** chemotherapy, gastrointestinal cancers, drug-related adverse effects, elderly, cancer

## Abstract

**Purpose:** Older cancer patients are more likely to develop and die from chemotherapy-related toxicity. However, evidence on drug safety and optimal effective doses is relatively limited in this group. The aim of this study was to develop a tool to identify elderly patients vulnerable to chemotherapy toxicity.

**Patients and methods:** Elderly cancer patients ≥60 years old who visited the oncology department of Peking Union Medical College Hospital between 2008 and 2012 were included. Each round of chemotherapy was regarded as a separate case. Clinical factors included age, gender, physical status, chemotherapy regimen and laboratory tests results were recorded. Severe (grade ≥3) chemotherapy-related toxicity of each case was captured according to the National Cancer Institute Common Terminology Criteria for Adverse Events, version 5.0. Univariate analysis was performed by chi-square statistics to determine which factors were significantly associated with severe chemotherapy toxicity. Logistic regression was used to build the predictive model. The prediction model was validated by calculating the area under the curve of receiver operating characteristic (ROC).

**Results:** A total of 253 patients and 1,770 cases were included. The average age of the patients was 68.9 years. The incidence of grade 3–5 adverse events was 24.17%. Cancer type (non-GI cancers), BMI<20 kg/m^2^, KPS<90%, severe comorbidity, polychemotherapy, standard dose chemotherapy, low white blood cells count, anemia, low platelet cells count, low creatine level and hypoalbuminemia were associated with severe chemotherapy-related toxicity. We used these factors to construct a chemotherapy toxicity prediction model and the area under the ROC curve was 0.723 (95% CI, 0.687–0.759). Risk of toxicity increased with higher risk score (11.98% low, 31.51% medium, 70.83% high risk; *p* < 0.001).

**Conclusion:** We constructed a predictive model of chemotherapy toxicity in elderly cancer patients based on a Chinese population. The model can be used to guide clinicians to identify vulnerable population and adjust treatment regimens accordingly.

## Introduction

Cancers are age-related diseases (Chang et al., 2019). According to the data on GLOBOCAN, approximately 50% of new diagnosed cancer patients in 2020 are elderly people over 65 years old (Sung et al., 2021). However, there are still many knowledge gaps in the treatment of elderly cancer patients (Hurria et al., 2014).

While older patients may respond similarly to anticancer treatments as younger patients, treatment-related toxicity remains a concern (Macchini et al., 2019). Studies have found that older patients are more likely to experience chemotherapy-related adverse events (Trumper et al., 2006; Muss et al., 2007; Asmis et al., 2008). Poor tolerability in the elderly population may be due to many factors, including age-related deterioration of multiple organ functions, comorbidities, polypharmacy, and other problems that can lead to altered pharmacokinetics and pharmacodynamics of chemotherapy drugs (Brunello et al., 2009; Feliu et al., 2018).

Dose reduction as a strategy to improve patient tolerability while preserving the antitumor effect has been identified as a promising approach (Hall et al., 2021). However, the elderly population is a highly heterogeneous group, with chronological age often not reflecting functional status and chemotherapy tolerance (Hernandez Torres and Hsu, 2017). Moreover, current guidelines for cancer treatment primarily rely on evidence obtained from clinical trials, which often exclude older patient population (Joharatnam-Hogan et al., 2020). Therefore, more evidence is needed to identify elderly populations at risk and guide adjustments of antitumor drug doses.

Several predictive tools have been developed to assess the risk of chemotherapy toxicity, including the Cancer and Aging Research Group (CARG) score and the Chemotherapy Risk Assessment Scale for High-Age Patients (CRASH) Score (Hurria et al., 2011; Extermann et al., 2012). However, it should be noted that these models were primarily based on data from the Caucasian population and have limited applicability to Asian populations.

In this article, we collected data of elderly cancers patients from a tertiary hospital in inland China and analyzed the incidence of severe chemotherapy-related adverse events. We aimed at predicting the risk of chemotherapy toxicity using a logistic regression model and this predictive model should give more suggestion when discussing the risks and benefits of chemotherapy with older adults.

## Methods

### Setting and patient

This study retrospectively analyzed elderly cancer patients who attended Peking Union Medical College Hospital between 2008 and 2012. Patient data were extracted, encrypted, and de-identified by 2 professional researchers in 2013. The Ethics Committee at Cancer Hospital, Chinese Academy of Medical Sciences and Peking Union Medical College approved the study and waived the need for patient consent because of the retrospective design of this study.

1,453 cancer patients were reviewed, and 253 patients were enrolled. The inclusion criteria were patients (1) who were older than or equal to 60 years; (2) with a clear pathological diagnosis of malignancy or lymphoma; and (3) receiving at least one chemotherapy treatment. Patients whose diagnosis was unclear or who did not receive chemotherapy were excluded.

Each chemotherapy cycle received by each patient is considered as a separate case in our study. In total, 1,770 cases are included.

### Primary outcome

The primary endpoint was the occurrence of severe hematologic and non-hematologic chemotherapy-related toxicity (grade 3 [hospitalization indicated], grade 4 [life threatening], and grade 5 [treatment-related death]), graded using National Cancer Institute Common Terminology Criteria for Adverse Events (NCI CTCAE) v5.0 criteria (National Cancer Institute, 2017). This endpoint was chosen because most guidelines recommend dose adjustment when severe toxicity (grade≥ 3) occurs.

### Data collection

Patient demographic data (gender and age), tumor-related conditions (type and stage), body mass index (BMI), and comorbidities were collected. The Charlson comorbidity index (CCI) score was used to assess the severity of comorbidity (Charlson et al., 1987). In calculating the CCI score, metastatic solid tumor was excluded as comorbid conditions, given that our data itself is a cohort of tumor patients and most of them had distant metastases.

Before each chemotherapy cycle, the following data were captured: (1) Chemotherapy regimen, number of chemotherapy drugs, and chemotherapy doses. (2) Eastern Cooperative Oncology Group (ECOG) Performance Status Scale and Karnofsky score (KPS). (3) Laboratory variables included but are not limited to white blood cells, hemoglobin, platelets, transaminases, creatinine, and albumin.

### Statistical analyses

We performed descriptive statistics of chemotherapy-related adverse events in all patients and calculated the incidence of hematological and non-hematological toxicities.

### Model development

First, we used chi-square (χ^2^) tests to identify variables associated with grade 3–5 chemotherapy-related adverse events. Variables included age, gender, tumor type (non-gastrointestinal cancers or gastrointestinal cancers), stage (≤3 or 4), number of chemotherapy drugs (single or multiple drugs), chemotherapy doses (reduced or standard doses), BMI (<20 kg/m^2^ or ≥20 kg/m^2^), ECOG (>1 or ≤1), KPS (<90% or ≥90%), comorbidity score (CCI≥4 or <4) and multiple laboratory variables.

Variables with *p*-values less than 0.1 and certain clinical variables strongly associated with the outcome would be selected as model factors. We established the predictive model by a multivariate logistic regression model. The Youden Index (Youden, 1950) was used to identify the cut point with the highest sensitivity and specificity when classifying the presence or absence of toxicity. The discrimination of models was evaluated by calculating the area under the receiver operating characteristic (ROC) curve.

### Developing the scoring system

A risk score for each risk factor was calculated by dividing the coefficient of the variable by the lowest coefficient in the model, rounded to the nearest 0.5 times. (Concato et al., 1993; Walter et al., 2001). After that, the sum of scores for each chemotherapy case was calculated. The sample was divided into three risk strata (low, medium, and high risk) based on approximate quartiles of risk score with the middle two quartiles combined. The difference in toxicity incidence among the strata was evaluated by χ^2^ test.

### Model validation

The model was internally validated. We obtain the area under the ROC curve (AUC) of the model. If the AUC is larger than 0.7, it means the model is valid. All statistical analyses were performed by using SPSS.

## Results

### Characteristics of patients and cases

The basic information of the patients was listed in Table1. Male patients accounted for 66.4% and female patients accounted for 33.6%. The average age of patients was 68.9 years old. More than 75% of patients were older than 65 years old, but the proportion of the oldest old patients (≥80 years old) was relatively small, accounting for only about 5%. Staging IV account for 43.48%. The most common cancer type was gastrointestinal cancer (172 cases, about 67.98% of 253 patients). Among GI cancer, gastric cancer accounted for about 20% (34 cases) and colorectal cancer accounted for about 45% (78 cases). Among non-GI tumors, lung cancer accounted for the largest proportion (56 cases, 70%). As for patients’ treatments, 18.18% of patients experienced 1–3 rounds of chemotherapy, 28.46% experienced 4-6 rounds, and 53.36% experienced more than 7 rounds of chemotherapy. 76.28% of patients had none or less severe comorbidity (CCI <4), whereas 23.72% of patients had severe comorbidity (CCI≥ 4). The most common comorbidities were cardiovascular disease, diabetes mellitus and chronic respiratory disease, consistent with previous surveys of chronic disease burden in the elderly population (Supplementary Figure S1) (Prince et al., 2015).

**TABLE 1:** Patients characteristics.

Characteristics	No. of patients	% Patients
**Baseline Characteristics (N = 253)**		
Gender		
Male	168	66.40
Female	85	33.60
Age, years (Average: 68.9)		
60–64	63	24.90
65–69	75	29.65
70–74	58	22.92
75–79	43	17.00
≥80	14	5.53
Cancer type		
GI	172	67.98
Esophageal Cancer	7	4.07
Gastric Cancer	34	19.77
Colorectal Cancer	78	45.35
Pancreatic Cancer	17	9.88
Bile Duct Cancer	1	0.58
Liver Cancer	2	1.16
Non-GI	80	31.62
Genitourinary Cancer	8	10.00
Lung Cancer	56	70.00
Lymphoma	8	10.00
Melanoma	3	3.75
Miss	1	0.40
Cancer stage		
0-III	113	44.66
IV	110	43.48
Miss	30	11.86
Chemotherapy Cycle		
1–3	46	18.18
4–6	72	28.46
7+	135	53.36
Comorbidity		
None or less severe	193	76.28
Severe	60	23.72
**Available Characteristics of Cases**		
KPS (%) (n = 1,429)		
≥90	1,103	77.19
< 90	326	22.81
ECOG (n = 1,470)		
≤1	1,336	90.88
>1	134	9.12
BMI, kg/m^2^ (n = 1,490)		
<18	151	10.13
[18, 24)	865	58.05
[24, 28)	372	24.97
≥28	102	6.85
Numebr of chemotherapy agents (n = 1,757)		
1	336	19.12
≥2	1,421	80.88
Dose (n = 1,489)		
Reduced	751	50.44
Standard	738	49.56

Note: GI, gastrointestinal cancer.

Most patients had relatively good physical status during each chemotherapy cycle: Among the 1,429 cases, which KPS scores were available, 77.19% of patients (1,103 cases) had a KPS score≥ 90%. Among the 1,470 cases, which ECOG scores were available, 90.88% (1,336 cases) had an ECOG score ≤1. More than half of the cases (58.05%, 865 cases from total 1,490 valid BMI records) had normal weight, 31.82% (474 cases) were overweight or obese (BMI >24 and 28 kg/m^2^, based on the new Chinese criteria (Pan et al., 2021)) and 10.13% (151 cases) were underweight (BMI <18 kg/m^2^). Besides, in 1757 total valid data of the number of chemotherapy agents, approximately 80% of cases were treated with a multidrug chemotherapy regimen and about 20% were treated with single-agent chemotherapy. In detail, 56.55% of the regimen contained fluoropyrimidine (such as capecitabine, fluorouracil and tegafur), 60.28% contained platinum, and about 20% contained taxanes (Supplementary Table S1 **)**. In addition, in 1,489 total valid data of chemotherapy dose, 50.44% of the patients received physician-determined reduced-dose chemotherapy, while the others used the guideline-recommended standard dose. Of patients who underwent dose adjustment, more than half of the patients (64.85%) received chemotherapy with a reduction of 20%–35%, 29.69% with a reduction of 35%–50%, and only 5.46% with a reduction of more than 50% (Supplementary Table S2).

### Chemotherapy-related toxicity

Consistent with previous studies (Trumper et al., 2006; Muss et al., 2007; Asmis et al., 2008), the incidence of chemotherapy-related toxicity in elderly tumor patients was high, with 79.72% of patients experiencing any grade of adverse events, of which about 24.16% were grade 3–4 adverse events (Table 2). 12 patients died but were considered not to be directly related to chemotherapy toxicity.

**TABLE 2:** Treatment-related adverse events.

	Cases		Severe toxicity	
	No.	%	No.	%
**Non-hematologic**				
Weakness	257	14.52	6	2.33
Weight loss	80	4.52	12	5.00
Rash	79	4.46	3	3.80
Alopecia	31	1.75	0	0
Fever	93	5.25	4	4.3
Infection	21	1.19	6	28.57
Muscle Pain	48	2.71	0	0
Headache and Dizziness	56	3.16	0	0
Insomnia	46	2.60	0	0
Cough	30	1.69	0	0
Dyspnea	47	2.66	1	2.13
Nausea	398	22.49	8	2.01
Vomiting	200	11.30	7	3.50
Lack of Appetite	376	21.24	7	1.86
Diarrhea	186	10.51	12	6.45
Constipation	171	9.66	0	0
Abdominal Pain and Bloating	127	7.18	0	0
Other Gastrointestinal Disorders	57	3.22	0	0
Neurotoxicity	129	7.29	1	0.78
Edema	59	3.33	0	0
Thromboembolic Event	22	1.24	0	0
ALT Elevation (N = 654)	66	10.09	3	4.55
Abnormal Total Bilirubin (N = 637)	89	13.97	0	0
Creatinine Increased (N = 650)	42	6.46	0	0
Hypokalemia (N = 583)	61	10.46	0	0
Hypoalbuminemia (N = 559)	120	21.47	0	0
**Hematologic**				
White blood cell count decreased (N = 818)	578	70.66	118	20.42
Anemia (N = 900)	576	64.00	46	7.99
Neutrophil count decreased (N = 918)	421	45.86	150	35.63
Platelet count decreased (N = 909)	306	33.66	55	17.97
**Total**				
Total Adverse Events (N = 1770)	1,411	79.72	341	24.17

Note: Severe toxicity refers to grade 3–5 toxicity, defined by National Cancer Institute Common Terminology Criteria for Adverse Events (NCI CTCAE) v5.0 criteria.

Among all non-hematologic adverse events in total 1770 cases, nausea (398 [22.49%]), lack of appetite (376 [21.24%]), hypoalbuminemia (120 [21.47%]) and weakness (257 [14.52%]) were most common, but mostly to a lesser extent. Although a low proportion of the overall cases, infection was the most common severe non-hematologic adverse events: In 1770 cases, only 21 cases had infection but 6 of them (1.19%) were grade 3–5 toxicity, which was the highest proportion of grade 3–5 toxicity among all non-hematologic adverse events, accounting for 28.57%.

The incidence of hematological toxicity was higher than non-hematological toxicity. Among valid data: The most common hematologic adverse events were white blood cell decreased (578 [70.66%]), followed by anemia (576 [64%]) and thrombocytopenia (306 [33.66%]). Among severe hematologic adverse events, neutropenia was the most common (150 [35.63%]).

### Chemotherapy-related toxicity predict model

We assessed the association between severe chemotherapy-related toxicity (Grade≥ 3) and multiple clinical variables (Table 3). There are 12 variables significantly associated with severe chemotherapy-related toxicity: cancer type (non-GI, *p* < 0.001), number of chemotherapy agents (polychemotherapy, *p* = 0.042), chemotherapy dose (standard dose, *p* = 0.078), BMI (<20 kg/m^2^, *p* < 0.001), KPS (<90%, *p* < 0.001), ECOG (>1, *p* = 0.036), comorbidity (CCI≥4, *p* = 0.002), low white blood cell (<4 × 10^9^/L, *p* < 0.001), low neutrophils (<2×10^9^/L, *p* < 0.001), anemia (hemoglobin<110 g/L, *p* < 0.001), low platelets (<100×10^9^/L, *p* < 0.001), hypoalbuminemia (albumin<35 g/L, *p* < 0.001), and low creatine level (<59 μmol/L, *p* < 0.001). Outliers in these factors can significantly increase the probability of severe chemotherapy-related toxicity. Focusing on BMI, for example, of the 1,176 non-severe toxicity cases, the proportion of BMI<20 kg/m^2^ is 20.07% (236 cases), which is increasing significantly to 28.66% (90 cases) in 314 cases with severe toxicity. At the same time, the chi-square (χ^2^) test obtained *p* < 0.001, which showed that lower BMI was significantly positively correlated with the occurrence of severe chemotherapy-related toxicity.

**TABLE 3:** Association between case characteristics and toxicity.

Variable	Cases		Non-severe toxicity		Severe toxicity		*p*-Value
	No.	%	No.	%	No.	%	
**Demographics**							
Gender							
	1,770		1,417	80.06	353	19.94	0.118
Male	1,146	64.75	930	65.63	216	61.19	
Female	624	35.25	487	34.37	137	38.81	
Age, years							
	1,770		1,417	80.06	353	19.94	0.136
< 69	920	51.98	724	51.09	196	55.52	
≥69	850	48.02	693	48.91	157	44.48	
**Tumor and treatment**							
Cancer type							
	1,768		1,415	80.03	353	19.97	<0.001
GI	1,343	75.96	1,126	79.58	217	61.47	
non-GI	425	24.04	289	20.42	136	38.53	
Number of chemotherapy agents							
	1,757		1,410	80.25	347	19.75	0.042
1	336	19.12	283	20.07	53	15.27	
≥2	1,421	80.88	1,127	79.92	294	84.73	
Dose							
	1,489		1,174	78.84	315	21.16	0.078
Reduced	751	50.44	606	51.62	145	46.03	
Standard	738	49.56	568	48.38	170	53.97	
Cancer stage							
	1,607		1,309	81.46	298	18.54	0.834
I ∼ III	730	45.43	593	45.30	137	45.97	
IV	877	54.57	716	54.70	161	54.03	
**Geriatric assessment**							
KPS, %							
	1,429		1,120	78.38	309	21.62	<0.001
≥90	1,103	77.19	896	80	207	67	
<90	326	22.81	224	20	102	33	
BMI, kg/m^2^							
	1,490		1,176	78.93	314	21.07	<0.001
≥20	1,164	78.12	940	79.93	224	71.34	
<20	326	21.88	236	20.07	90	28.66	
Comorbidity							
	1,770		1,417	80.06	353	19.94	0.002
None or less severe	1,353	76.44	1,105	77.98	248	70.25	
severe	417	23.56	312	22.02	105	29.75	
ECOG							
	1,470		1,167	79.39	303	20.61	0.036
≤1	1,336	90.88	1,070	91.69	266	87.79	
>1	134	9.12	97	8.31	37	12.21	
**Laboratory variables**							
White blood cell, $\times 10^{9}/L$							
	1,580		1,261	79.81	319	20.19	<0.001
≥4	1,160	73.42	963	76.37	197	61.76	
<4	420	26.58	298	23.63	122	38.24	
Hemoglobin, g/L							
	1,579		1,260	79.80	319	20.20	<0.001
≥110	1,103	69.85	928	73.65	175	54.86	
<110	476	30.15	332	26.35	144	45.14	
Neutrophils, $\times 10^{9}/L$							
	1,559		1,247	79.99	312	20.01	<0.001
≥2	1,176	75.43	971	77.87	205	65.71	
<2	383	24.57	276	22.13	107	34.29	
Platelets, $\times 10^{9}/L$							
	1,574		1,256	79.80	318	20.20	<0.001
≥100	1,362	86.53	1,115	88.77	247	77.67	
<100	212	13.47	141	11.23	71	22.33	
Creatine level, μmol/L							
	1,500		1,195	79.67	305	20.33	<0.001
≥59	1,192	79.47	974	81.51	218	71.48	
<59	308	20.53	221	18.49	87	28.52	
Albumin, g/L							
	1,300		1,034	79.54	266	20.46	<0.001
≥35	1,127	86.69	915	88.49	212	79.70	
<35	173	13.31	119	11.51	54	20.30	
Creatine clearance, Cockcroft-Gault, mL/min							
	1,382		1,087	78.65	295	21.35	0.438
≥60	1,078	78	843	77.55	235	79.66	
<60	304	22	244	22.45	60	20.34	
ALT, U/L							
	1,500		1,195	79.67	305	20.33	0.92
≤40	1,423	94.87	1,134	94.90	289	94.75	
>40	77	5.13	61	5.10	16	5.25	

We selected 11 variables to construct a chemotherapy-related toxicity predictive model (Table 4). The variables included cancer type (non-GI cancer), BMI< 20 kg/m^2^, KPS< 90%, severe comorbidity, polychemotherapy, standard dose chemotherapy and 5 laboratory variables (low white blood cells count, anemia, low platelet cells count, low creatine level and hypoalbuminemia.) Each variable was assigned a different risk score (ranged 1–3), with a total score of 21.

**TABLE 4:** Chemotherapy-related toxicity predictive model.

Toxicity type	Prevalent Cases (N = 1,088)		Severe Toxicity (N = 230)		Score
	No.	%	No.	%	
non-GI cancer	289	26.56	91	31.49	2.5
BMI<20 kg/m^2^	237	21.78	68	28.69	2
KPS<90%	250	22.98	74	29.60	2
Severe comorbidity	257	23.62	66	25.68	1.5
Polychemotherapy	928	85.29	198	21.34	1.5
Standard dose chemotherapy	553	50.83	112	20.25	1
WBC<4×10^9^/L	299	27.48	98	32.78	2.5
Hemoglobin<110 g/L	340	31.25	105	30.88	2
PLT <100×10^9^/L	141	12.96	57	40.43	3
Serum creatine <59 μmol/L	222	20.40	69	31.08	1.5
Albumin <35 g/L	149	13.69	47	31.54	1.5
Total score					21

### Model validation

**FIGURE 1:**
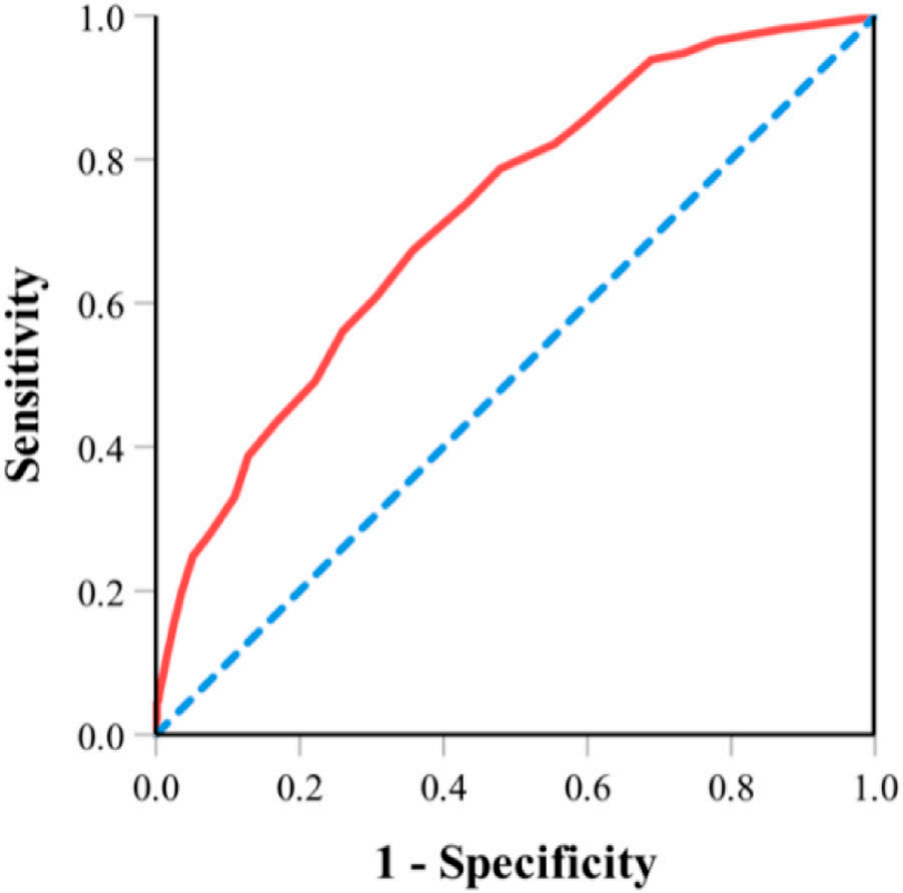
ROC curve for the predictive model. Note: Area under the ROC curve for the predictive model is 0.723 ([95% CI, 0.687 to 0.759]. ROC, receiver operating characteristic.

Risk score ranges from 0 to 21 points and was divided into three groups (low-risk group, 0 to 6 points; medium-risk group, 6.5 to 12 points; high-risk group, 12.5 to 21 points). Most patients (57.54%) were classified as low-risk group, 40.26% of patients were classified as medium-risk group, and 2.2% were classified as high-risk group. The risk of toxicity increased with increasing risk score (11.98% in the low-risk group, 31.51% in the medium-risk group, and 70.83% in the high-risk group; *p* < 0.001; Table 5). We examined the internal validation of this model: The area under the ROC curve for the predictive model is 0.723 ([95% CI, 0.687 to 0.759]. Figure 1), suggesting good predictive power of severe chemotherapy toxicity.

**TABLE 5:** Ability of risk score to predict chemotherapy toxicity.

Risk strata	Total case		Non-severe toxicity		Severe toxicity		*p*-Value	AUC
	No.	%	No.	%	No.	%		
By total score	1,088	100	858	78.86	230	21.14	<0.001	0.723
0–6 (low)	626	57.54	551	88.02	75	11.98		
6.5–12 (mid)	438	40.26	300	68.49	138	31.51		
12.5–21 (high)	24	2.20	7	29.17	17	70.83		

## Discussion

The tolerance of chemotherapy in elderly cancer patients is a concern. Predicting the risk of chemotherapy toxicity in advance can help clinicians identify vulnerable populations.

Several models have been developed to predict chemotherapy toxicity. In 2011, Hurria et al. constructed the CARG score, which is a predictive tool and a risk stratification schema that aims to identify older adults at low, intermediate, or high risk of chemotherapy toxicity (Hurria et al., 2011). The variables included in the model are age, tumor type, treatment intensity, laboratory test values, and a 5-question brief geriatric assessment. The CARG tool is simple to use and has been validated in many studies (Kotzerke et al., 2019; Zhang et al., 2019; Ostwal et al., 2021). In the same year, Extermann et al. published the CRASH Score, which is more comprehensive but relatively complex to use. Among Asian populations (Extermann et al., 2012), the Korean Cancer Study Group (KCSG) score have been developed by Kim and his colleagues but has not yet been widely used (Kim et al., 2018).

In this retrospective single-center study, we sought to develop an objective predictive model based on a Chinese population cohort. In our model, we included tumor types (non-GI cancers), BMI, KPS, severe comorbidity, chemotherapy regimens (multidrug and standard dose chemotherapy) and 5 laboratory variables to predict the risk of chemotherapy-related toxicity.

The effect of BMI, KPS or ECOG score, comorbidity and chemotherapy regimens on chemotherapy tolerance have been widely discussed ((Hurria et al., 2011; Lee et al., 2011; Extermann et al., 2012; Dotan et al., 2020).

Among the laboratory variables, anemia is frequently diagnosed in elderly people and is associated with reduced overall survival. (Knight et al., 2004; Stauder et al., 2018). Anemia leads to increased serum free concentrations of many chemotherapeutic agents that need to bind to red blood cells, thereby increasing toxicity. (Schrijvers et al., 1999; Feliu et al., 2018). Similarly, hypoalbuminemia, an indicator of malnutrition, increases the serum concentration of some drugs and leads to increased chemotherapy toxicity. (Schrijvers et al., 1999; Feliu et al., 2018).

The association between malnutrition and chemotherapy tolerance has been demonstrated in many studies. (Arrieta et al., 2010; Barret et al., 2011; Bozzetti, 2017). Low serum creatinine is a marker of reduced muscle mass associated with malnutrition, aging, and chronic disease, but is often overlooked in clinical practice (Cartin-Ceba et al., 2007; de Jong et al., 2022). In our cohort, there is a large proportion of patients (308/1,500) had a lower-than-normal serum creatinine level which is associated with high risk of chemotherapy-related toxicity. We also analyzed the effect of elevated serum creatinine level on the risk of toxicity but did not obtain statistically significant results, probably because of the small number of this group of people.

Interestingly, non-GI cancer type is associated with severe toxicity in our cohort, which is the opposite of the CARG study results. In the CARG study, patients with gastrointestinal (GI) or genitourinary (GU) tumors had a higher risk of chemotherapy toxicity (Hurria et al., 2011). We thought this may be related to the different toxicity profiles between different human races. Fluoropyrimidine drugs such as 5-fluorouracil, S-1 and capecitabine are recommended by many guidelines and frequently used in the treatment of GI cancers. (Park and Chun, 2013). Studies have found that fluoropyrimidine drugs cause a higher incidence of severe gastrointestinal toxicity in Caucasians than in Asians, partly because of polymorphic differences in the CYP2A6 gene. (Ajani et al., 2005; Haller et al., 2008; Chuah et al., 2011; Ma et al., 2012).

Notably, in our data, the risk of chemotherapy toxicity was not increased with age. This may be because half of our patients had already received dose-reduced regimen. In clinical practice, oncologists often reduce the doses for elderly and frail patients based on their clinical assessment to prevent severe toxicity. (Gajra et al., 2015). However, it is currently unknown whether the empirical adjustment of drug doses will result in optimal clinical outcomes. This uncertainty prompted the development of this predictive model to assess the risk of chemotherapy toxicity in older patients.

There are some limitations to this study. First, this study was a retrospective single-center analysis and more external validation is needed. A prospective clinical study is underway at our center, and further data is expected to confirm the utility of this predictive model.

Second, we did not include comprehensive geriatric assessments such as functional capabilities, cognitive status, emotional status, and social support. Although the ECOG score, comorbidity score, and laboratory variables can partially reflect the health status of elderly patients, they cannot replace the comprehensive geriatric assessment (CGA). However, the comprehensive geriatric assessment is still rarely used in China. In a recent study, the use of CGA tools was found to be only 56.9% in tertiary hospitals in China. (Wu et al., 2022). We are actively collaborating with geriatricians to introduce geriatric assessments into our center and plan to incorporate additional CGA components in future iterations of this model.

This study has some future directions. Many novel anti-tumor therapies such as targeted therapy, immunotherapy, and CAR-T therapy have emerged. However, there are less evidence on the use of these drugs in elderly patients. Toxicity prediction models should also be constructed and validated for these treatments. In addition, even though we successfully stratified patients according to their risk of chemotherapy toxicity, it is still unclear what percent of dose reduction should be applied to each group of patients.

In conclusion, we constructed a simple and objective model with 11 variables to predict chemotherapy-related toxicity in elderly cancer patients. This model aims to help clinicians identify vulnerable populations as well as formulate the best treatment and nursing strategies for elderly cancer patients.

## Data Availability

The raw data supporting the conclusion of this article will be made available by the authors, without undue reservation.
