# Supplementary material for: Predictive model of chemotherapy-related toxicity in elderly Chinese cancer patients
Source: Front Pharmacol. 2023 Apr 26;14:1158421. doi: 10.3389/fphar.2023.1158421 (PMC10169599; doi:10.3389/fphar.2023.1158421)
Supplement: Supplementary file 1 [file Table1.pdf]

# *Supplementary Material*

**Supplementary table 1 Chemotherapy regimen.**

| Regimen | No. | % |
|---|---|---|
| Fluoropyrimidine-based | 1001 | 56.55% |
| Platinum-based | 1067 | 60.28% |
| Taxanes-based | 327 | 18.47% |
