# Supplementary material for: Predictive model of chemotherapy-related toxicity in elderly Chinese cancer patients
Source: Front Pharmacol. 2023 Apr 26;14:1158421. doi: 10.3389/fphar.2023.1158421 (PMC10169599; doi:10.3389/fphar.2023.1158421)
Supplement: Supplementary file 2 [file Table2.pdf]

Supplementary Material

Supplementary Table 2 Dose adjustment in dose reduction group.

Dose reduction	No.	%
25~30%	487	64.85
30~50%	223	29.69
>50%	41	5.46
Total	751	
