# Supplementary material for: Predictive model of chemotherapy-related toxicity in elderly Chinese cancer patients
Source: Front Pharmacol. 2023 Apr 26;14:1158421. doi: 10.3389/fphar.2023.1158421 (PMC10169599; doi:10.3389/fphar.2023.1158421)
Supplement: Supplementary file 3 [file Image1.pdf]

Supplementary Material

Supplementary figure 1 Distribution of comorbidity.

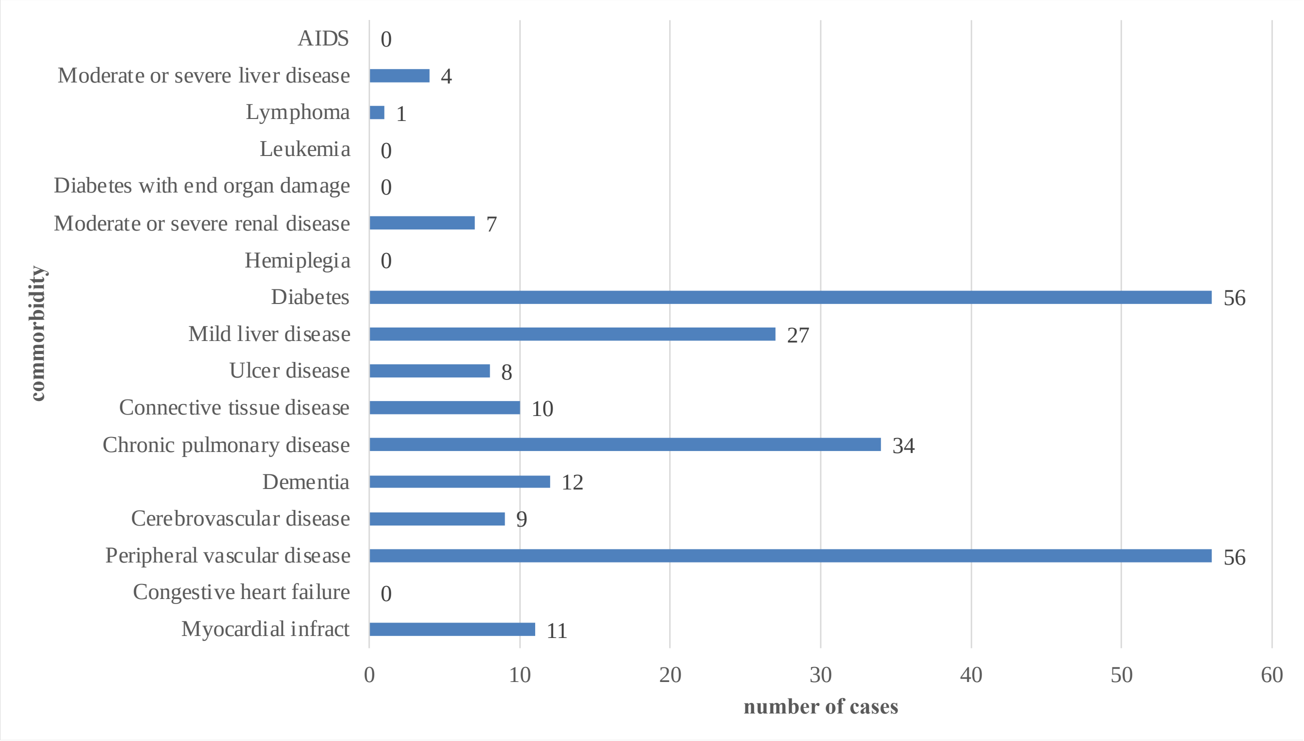

Note: Comorbidity of enrolled patients (n=286). Only comorbidities included in the Charlson Comorbidity Index were collected.
